# *TNFRSF13B* genotypes control immune-mediated pathology by regulating the functions of innate B cells

**DOI:** 10.1172/jci.insight.150483

**Published:** 2021-09-08

**Authors:** Mayara Garcia de Mattos Barbosa, Adam R. Lefferts, Daniel Huynh, Hui Liu, Yu Zhang, Beverly Fu, Jenna Barnes, Milagros Samaniego, Richard J. Bram, Raif S. Geha, Ariella Shikanov, Eline T. Luning Prak, Evan A. Farkash, Jeffrey L. Platt, Marilia Cascalho

**Affiliations:** 1 Department of Surgery,; 2 Department of Pathology, and; 3 Department of Medicine, University of Michigan, Ann Arbor, Michigan, USA.; 4 Department of Pediatric and Adolescent Medicine and Department of Immunology, Mayo Clinic, Rochester, Minnesota, USA.; 5 Division of Immunology, Boston Children’s Hospital, Boston, Massachusetts, USA.; 6 Department of Pediatrics, Harvard Medical School, Boston, Massachusetts, USA.; 7 Department of Biomedical Engineering, University of Michigan, Ann Arbor, Michigan, USA.; 8 Department of Pathology and Laboratory Medicine, Perelman School of Medicine, University of Pennsylvania, Philadelphia, Pennsylvania, USA.; 9 Department of Microbiology and Immunology, University of Michigan, Ann Arbor, Michigan, USA.

**Keywords:** Genetics, Inflammation, Genetic variation, Immunoglobulins, Mouse models

## Abstract

Host genes define the severity of inflammation and immunity but specific loci doing so are unknown. Here we show that TNF receptor superfamily member 13B (*TNFRSF13B*) variants, which enhance defense against certain pathogens, also control immune-mediated injury of transplants, by regulating innate B cells’ functions. Analysis of *TNFRSF13B* in human kidney transplant recipients revealed that 33% of those with antibody-mediated rejection (AMR) but fewer than 6% of those with stable graft function had *TNFRSF13B* missense mutations. To explore mechanisms underlying aggressive immune responses, we investigated alloimmunity and rejection in mice. Cardiac allografts in *Tnfrsf13b-*mutant mice underwent early and severe AMR. The dominance and precocity of AMR in *Tnfrsf13b*-deficient mice were not caused by increased alloantibodies. Rather, *Tnfrsf13b* mutations decreased “natural” IgM and compromised complement regulation, leading to complement deposition in allografted hearts and autogenous kidneys. Thus, WT *TNFRSF13B* and *Tnfrsf13b* support innate B cell functions that limit complement-associated inflammation; in contrast, common variants of these genes intensify inflammatory responses that help clear microbial infections but allow inadvertent tissue injury to ensue. The wide variation in inflammatory reactions associated with *TNFRSF13B* diversity suggests polymorphisms could underlie variation in host defense and explosive inflammatory responses that sometimes enhance morbidity associated with immune responses.

## Graphical abstract

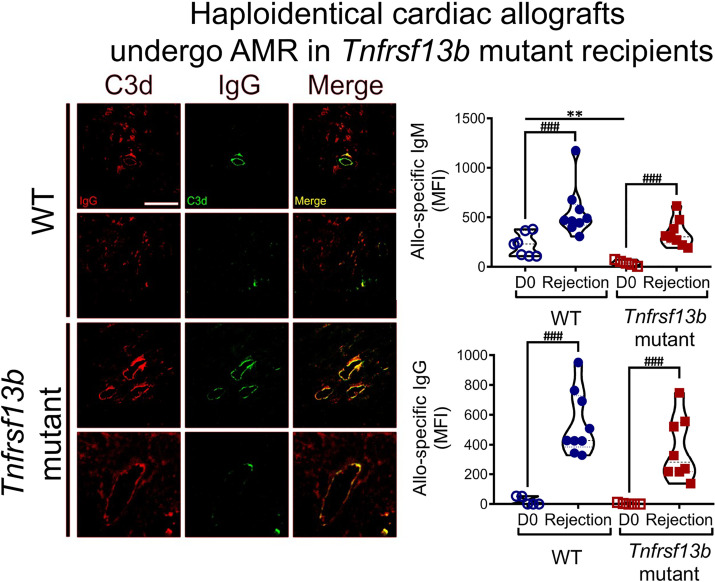

## Introduction

Immunity and tolerance are governed at least in part by highly polymorphic genes of the MHC (1, 2), first appreciated as inherited determinants of the ability to produce antibodies against and reject foreign cells and tissues (3, 4). Yet, decades of experience in clinical transplantation reveals an inexact relationship between production of antibodies against foreign MHC and antibody-mediated rejection (AMR) of transplants expressing that MHC (5, 6). MHC also governs the ability to mount antibody responses to pathogens, which promote resistance and immunity in some (7) but appear to promote “autoimmune” pathology and explosive inflammatory reactions in others (8). Having recently discovered that a highly polymorphic gene (TNF receptor superfamily member 13B, *TNFRSF13B*) remote from MHC determines the character of primary immunity to enteric pathogens and whether immunity confers protection (9), we wondered whether and how variants of that gene could explain the not uncommon dissociation between transplant immunity and rejection of transplants and more broadly whether polymorphism at this locus could explain profoundly divergent impact of immunity and inflammation.

There are theoretical reasons to think that the *TNFRSF13B* genotype might influence the character and outcome of immunity (10). *TNFRSF13B* is among the most polymorphic genes in humans and other mammalian species. *TNFRSF13B* variants appear to have been under positive selection (11–13); MHC variants, in contrast, appear to have been under moderate purifying pressure (14). The protein encoded by *TNFRSF13B*, the transmembrane activator calcium modulator and cyclophilin ligand interactor (TACI), governs T cell–independent antibody responses (15) and the maturation of and selection of T cell–dependent B cell responses (9). *TNFRSF13B* could thus govern the balance between immunity and tolerance (16).

To examine whether *TNFRSF13B* polymorphisms could weigh on immune-mediated pathology, we asked whether missense mutations in human subjects segregate with the antibody-mediated injury in kidney transplants and whether and how similarly disruptive genotypes in mice predispose to antibody-mediated injury in allografts. Our findings both in humans and in mice reveal a clear association between *TNFRSF13B* genotype and the propensity of antibody responses to trigger alloimmune pathology. While this association might reflect several functions of *TNFRSF13B*, we show in mice the propensity is owed to the functions of innate B cells. That the extraordinary polymorphism of *TNFRSF13B* has been maintained across mammalian species likely suggests the aggressive, highly inflammatory responses confer host defense; however, our results also show this benefit is balanced by the risk that immunity will eventuate in unbridled inflammatory reactions severe enough to destroy an organ. Understanding the phenotypes accompanying *TNFRSF13B* alleles might thus offer new insight into the genetic basis of host defense and disease.

## Results

### TNFRSF13B missense mutations in human kidney transplant recipients.

Organ allografts commonly evoke B cell responses, leading to production of alloantibodies. Depending on the sensitivity of the assay used, alloantibodies are detected at one time or another in about half of kidney transplant recipients (17, 18). Despite the frequent detection of alloantibodies, fewer than 20% of those with such antibodies develop acute AMR, and occurrence is not necessarily related to the level or the isotype of alloantibodies (18–20). We reasoned that if functions imparted by *TNFRSF13B* were of consequence for the biological impact of antibody responses, the frequency of *TNFRSF13B* missense mutations might differ in transplant recipients with AMR and recipients free of rejection.

To examine that possibility, we sequenced by the Sanger method the 5 exons of *TNFRSF13B* in a cohort of human kidney transplant recipients that developed AMR (exon 1, *n* = 78; exon 2, *n* = 67; exon 3, *n* = 128; exon 4, *n* = 167; exon 5, *n* = 99) and in a cohort with stable graft function for up to 5 years after transplantation (exon 1, *n* = 84; exon 2, *n* = 110; exon 3, *n* = 119; exon 4, *n* = 115; exon 5, *n* = 102) (Table 1). We did not find any missense mutations on *TNFRSF13B* exons 1 and 2 in either group. Several nonsynonymous substitutions in exons 3, 4, and 5 are thought to perturb the TACI receptor function (11, 13, 21). More than 33% of those who developed AMR had missense mutations in exons 3 (C104R), 4 (A173T, A181E, K188del, K188M, G190R, S209F), and 5 (P251L), but fewer than 6% of the patients with stable graft function had these mutations (*P* < 0.0001). Eight missense mutations (including 1 in-frame deletion) in exons 3 and 4 were only present in kidney recipients who developed AMR. Only individuals with AMR had biallelic P251L missense mutations in exon 5, including one person with compound heterozygosity with 2 missense mutations on exon 4, A173T and K188M. Table 1 depicts the allele frequency of missense mutations in AMR and in stable graft function.

A summary of the expected impact of the variants on the function of the TACI receptor and in human health can be found summarized in Supplemental Table 1; supplemental material available online with this article; https://doi.org/10.1172/jci.insight.150483DS1 (22–26). These results suggest *TNFRSF13B* genotype might distinguish those who mount aggressive immunity to transplantation from those who do not; however, the degree of *TNFRSF13B* polymorphism in randomly bred populations and the many other potential variables would make it difficult to precisely connect a specific genotype with an outcome, much less with underlying mechanisms. We therefore explored the potential impact of *Tnfrsf13b* on the aggressiveness of immune responses in inbred mice.

### Tnfrsf13b and the rejection of organ transplants in mice.

**Figure 1:**
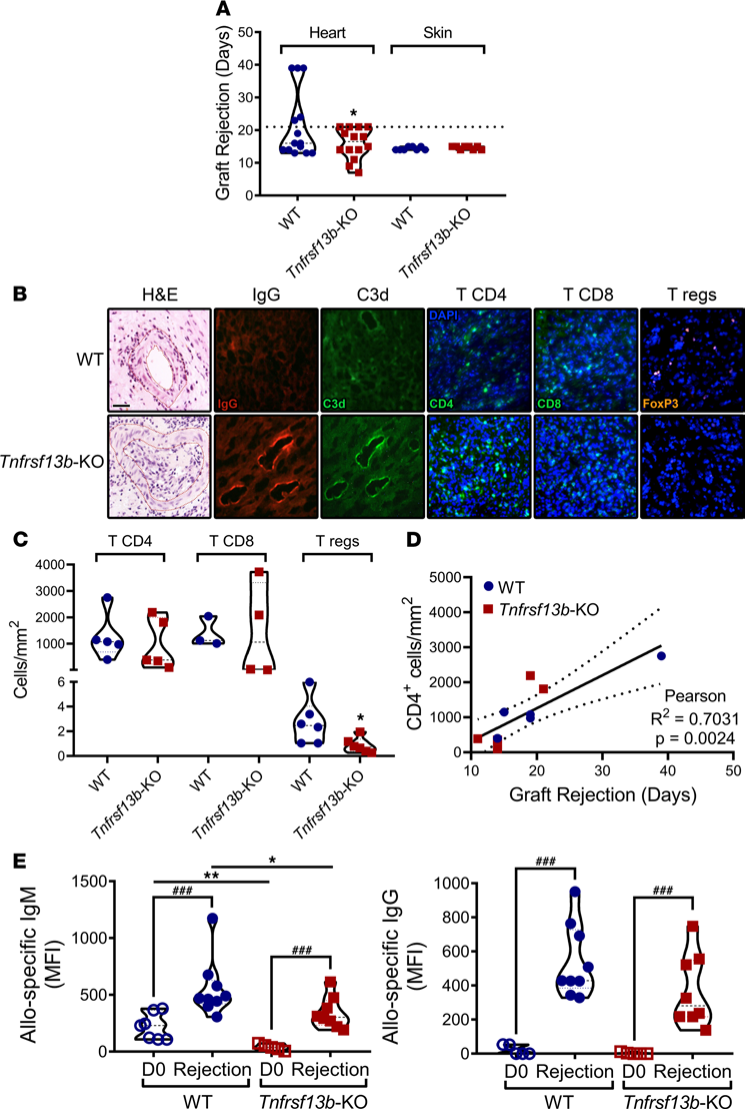
*Tnfrsf13b* deficiency evokes antibody responses to allografts and accelerates rejection of heart allografts. Hearts (transplanted heterotopically into the abdomen) and skin from CB6F1 mice (C57BL/6-BALB/c F1, H-2^b/d^ haplotype) were transplanted into C57BL/6 (H-2^b/b^ haplotype) WT and *Tnfrsf13b*-KO mice, and allograft survival was evaluated daily until rejection. (**A**) Graph represents the mean ± SEM of the number of days elapsed until allograft rejection (*n* = 14). See also Table 2. (**B**) H&E staining and anti-IgG, anti-C3d, anti-CD4, anti-CD8, and anti-Foxp3 (to detect T regulatory cells) immunostainings. Images are representative of a mouse of each group with viable heart excision at day 14 (H&E, C3d and IgG staining) or at rejection at day 19 (CD4, CD8, and Foxp3 staining). See also Supplemental Figure 1. Scale bar: 25 μm. (**C**) Graph depicts the number of CD4^+^, CD8^+^ of Foxp3^+^ (Tregs) T cells per mm^2^ in sections counted in 5 nonoverlapping fields obtained from grafts at rejection. (**D**) Graph represents the relationship between number of CD4^+^ T cells in graft sections and length of transplantation. Statistical analysis (Pearson correlation *P* = 0.0024) indicates that the number of CD4^+^ T cells/mm^2^ increases with time from transplantation. (**E**) Sera of recipient mice were collected before transplantation (day 0, D0) and at time of rejection, and allospecific IgM and IgG were analyzed by flow cytometry. Graphs are representative of mean ± SEM of 7–9 mice per group. One-tailed unpaired *t* test (**A**), 2-tailed unpaired *t* test (**C**), or 2-tailed paired and unpaired *t* tests or Mann-Whitney test (**E**): *P ≤ 0.05, **P ≤ 0.01 in relation to WT control; ^###^*P* ≤ 0.001 in relation to day 0 (D0).

To determine whether and how the *Tnfrsf13b* genotype could influence the level and/or aggressiveness of antigen-specific antibody responses, we compared the outcome of organ transplants placed in WT mice with the outcome in *Tnfrsf13b*-deficient mice. Hearts isolated from C57BL/6-BALB/c F1 (CB6F1) mice and transplanted heterotopically into C57BL/6 *Tnfrsf13b* WT mice contracted for 13–39 days (average = 21.2 days) whereas hearts transplanted in *Tnfrsf13b*-deficient mice contracted for 7–21 days (average = 15.9 days; *P* = 0.0136) (Figure 1A and Table 2). The most striking difference was not, however, in the length of graft function but instead the pathophysiology of allograft rejection. As expected, cardiac allografts in WT mice exhibited characteristic features of cell-mediated rejection suggested by the accumulation of CD4- and CD8-positive T cells in the graft and no deposits of IgM, IgG, or complement at 14 days posttransplantation (Figure 1B and Supplemental Figure 1). In contrast, the cardiac allografts in *Tnfrsf13b*-deficient mice contained abundant deposits of IgG and complement, features characteristic of AMR, as early as 14 days after transplantation (Figure 1B and Supplemental Figure 1). After 14 days features typical of cell-mediated rejection ensued in both *Tnfrsf13b*-deficient and WT recipients (Figure 1, B and C). T cell infiltration appeared to increase as a function of time from transplantation (Figure 1D). Furthermore, grafts in mutant mice had significantly fewer Tregs/mm^2^ than grafts in WT recipients (Figure 1, B and C). Consistent with a lack of impact on cellular immunity, skin allografts from CB6F1 were rejected as fast in *Tnfrsf13b*-KO as in WT recipients (Figure 1A and Table 2). Spontaneous AMR preceding cell-mediated lesions of organ transplants is quite unusual unless the recipient was previously sensitized to alloantigens or genetically manipulated in ways that profoundly increase production of alloantibodies (27). Since *Tnfrsf13b* deficiency decreases baseline production of antibodies and impairs maturation of B cell responses to antigen (28), the propensity toward development of AMR in *Tnfrsf13b*-deficient mice and possibly in humans with *TNFRSF13B* mutations would seem contrary to canonical functions of *TNFRSF13B*.

To determine whether disruption of *Tnfrsf13b* increases the magnitude of antibody response, accelerating rejection and causing dominance of antibody-mediated over cell-mediated pathology of allografts early in the graft’s life, we assayed alloantibodies in allograft recipients before transplantation and at the time of rejection. As Figure 1E shows, both WT and *Tnfrsf13b-*deficient mice mounted appreciable alloantibody responses after cardiac allotransplantation; however, the responses in *Tnfrsf13b*-deficient mice were less robust than responses in WT mice (IgM, *P* = 0.018; IgG, *P* = 0.057). Thus, the amount of alloantibody detected after transplantation would not appear to explain the dominance of antibody-mediated pathology in mutant recipients. Because nonsynonymous mutations in *TNFRSF13B* have been associated with autoantibodies, which could contribute to disease of the transplanted heart, we detected autoantibodies by staining the native hearts of both WT and *Tnfrsf13b*-deficient mice, also retrieved at rejection. Native hearts showed no deposition of IgM, IgG, or complement, indicating negligible autoantibody response in transplanted mice (Supplemental Figure 2).

**Figure 2:**
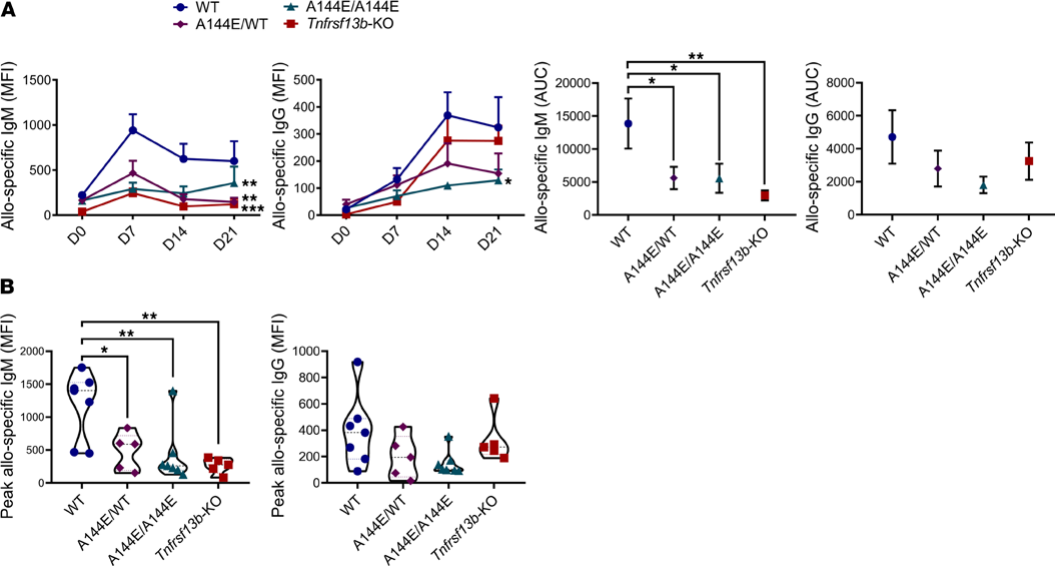
*Tnfrsf13b* genotype determines IgM responses to alloantigens. Mice were immunized by intraperitoneal injection with 5 × 10^7^ BALB/c splenocytes and thymocytes. Blood was collected weekly and levels of allospecific antibodies that bound BALB/c thymocytes were measured by flow cytometry pre- (day 0, D0) and postimmunization with allogeneic cells (days 7–21, D7, D14, D21). (**A**) Kinetics of allospecific IgM and IgG response were analyzed by flow cytometry. Curves were compared by 2-way repeated measures ANOVA followed by Dunnett’s multiple comparisons test. Area under the curve (AUC) was analyzed using the AUC function on GraphPad Prism 8. AUCs were compared using Brown-Forsythe ANOVA test followed by Dunnett’s multiple comparisons tests and by 2-tailed unpaired *t* tests with Welch’s correction (shown). (**B**) The peak concentrations of allospecific IgM and IgG in the blood are shown. Graphs are representative of mean ± SEM of 5–7 mice per group. Analysis was by 1-way ANOVA followed by Dunnett’s multiple comparisons tests. *P ≤ 0.05; **P ≤ 0.01; ***P ≤ 0.001 in relation to WT control.

Organ allografts can absorb a substantial fraction of alloantibody from serum, and hence it was possible that *Tnfrsf13b*-deficient mice produced more alloantibody than WT mice, but the increase was obscured by absorption. To exclude that possibility, we injected allogeneic splenocytes and thymocytes into the peritoneal cavity of WT mice and in *Tnfrsf13b*-deficient mice and assayed alloantibodies at various times thereafter. Because full *TNFRSF13B* deficiency is rarely observed in humans (16), we also tested the impact of monoallelic A144E missense mutations, which are homologous to a common human variant, A181E, that was detected in 2 transplant recipients with AMR (Table 1). As Figure 2 shows, monoallelic mutant, biallelic mutant, and *Tnfrsf13b*-deficient mice produced no more IgG alloantibodies and significantly fewer IgM alloantibodies than WT controls.

**Figure 3:**
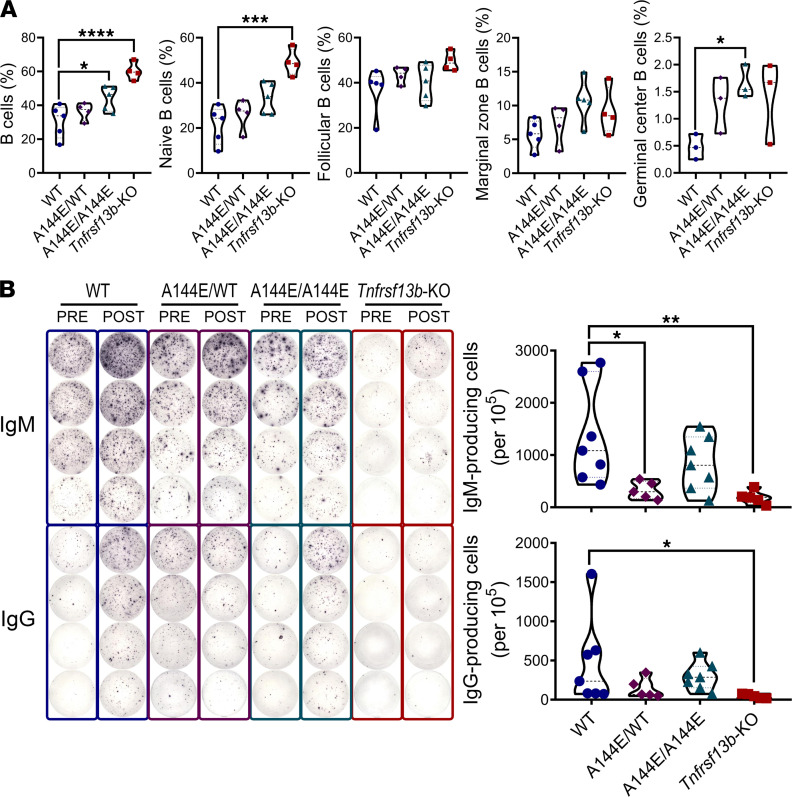
*Tnfrsf13b* deficiency impairs the differentiation of antibody-secreting cells in response to alloantigens. Mice were immunized by intraperitoneal injection with 5 × 10^7^ BALB/c splenocytes and thymocytes; spleens were harvested 10–21 days postimmunization. (**A**) Splenic percentages of live lymphocytes with phenotypes of B cells (CD19^+^) and naive B cells (CD19^+^IgD^+^) and percentages of CD19^+^ B cells that were marginal zone (CD19^+^CD21^hi^CD23^–^), follicular (CD19^+^CD21^+^CD23^+^) (at day 21), and germinal center (CD19^+^CD95^+^GL7^+^) B cells in the spleen (at day 10 after alloimmunization). (**B**) ELISPOT of IgM- and IgG-secreting cells pre- (day 0) and 21 days after alloimmunization. Graphs are representative of mean ± SEM of 3–7 mice per group 21 days after alloimmunization. One-way ANOVA or Brown-Forsythe and Welch’s ANOVA test with Dunnett’s multiple comparisons test: *P ≤ 0.05; **P ≤ 0.01; ***P ≤ 0.001; ****P ≤ 0.0001 in relation to the WT control.

To confirm that *Tnfrsf13b-*mutant mice do not mount more robust allospecific B cell responses, despite the propensity to inflict AMR on cardiac allografts, we enumerated the various types of B cells and the frequency of antibody-secreting cells in the spleens of WT and mutant mice before and after exposure to allogeneic splenocytes and thymocytes (respectively, Supplemental Figure 3 and Figure 3A). The immunized mutant mice had more total B cells and more B cells in marginal zones, follicles, and germinal centers (GCs) than WT mice (Figure 3A), consistent with increased response to activation (9, 28). However, relatively few of these B cells secreted antibody (Figure 3B). Clearly, some *Tnfrsf13b* missense mutations can underlie more aggressive B cell effector responses, possibly at the cost of inadvertent pathogenicity; however, neither the amplitude nor the pathogenicity is a simple function of the amount of antigen-specific IgG produced.

### Tnfrsf13b and the regulation of immunity.

**Figure 4:**
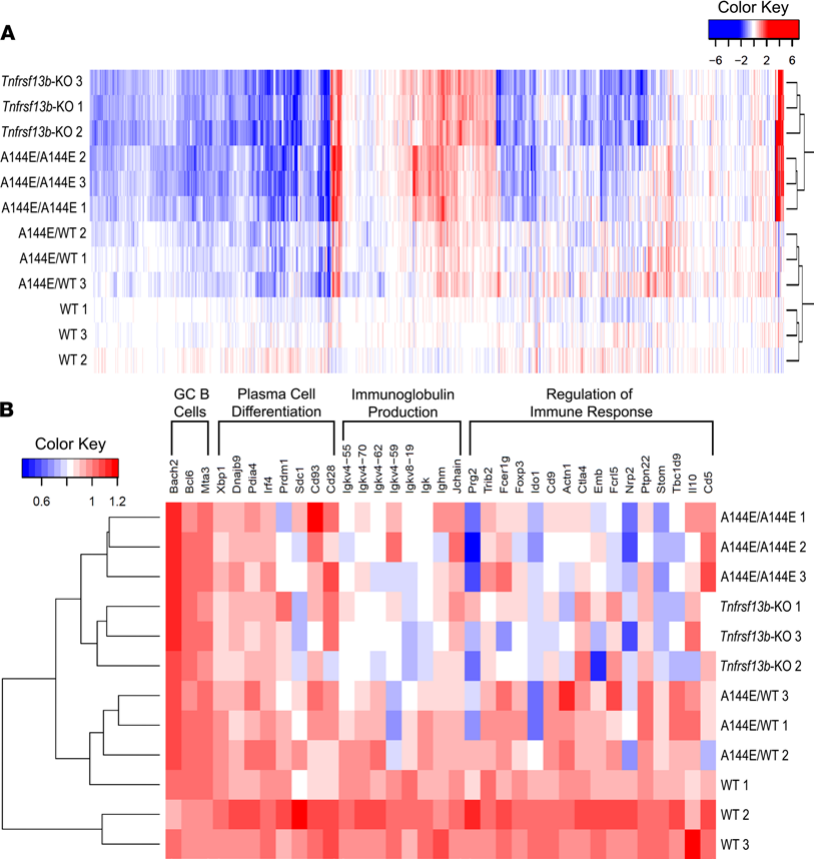
Microarray analysis of GC B cells of alloimmunized *Tnfrsf13b-*mutant mice. Mice with different *Tnfrsf13b* genotypes were immunized by intraperitoneal injection with 5 × 10^7^ BALB/c splenocytes and thymocytes. Spleens from immunized mice were collected 10 days later, and live CD19^+^CD95^+^GL7^+^ GC B cells were sorted. Gene expression was assessed by microarray (see also Supplemental Figure 4 and Supplemental Table 2). (**A**) Heatmap representing expression of genes found to be either significantly upregulated (red) or downregulated (blue) in the different *Tnfrsf13b* genotypes compared with WT. Each row represents 1 mouse (*n* = 3). (**B**) Expression of signature genes of GC B cells, plasma cells, and immunoglobulin production and regulation of immune response pathways found to be either upregulated (red) or downregulated (blue) by *Tnfrsf13b* deficiency (*n* = 3).

B cells exert functions besides production of antigen-specific IgG. Since IgG production is necessary but not sufficient to explain the dominance of the B cell effector responses in *Tnfrsf13b*-deficient or *Tnfrsf13b*-mutant mice (9), we wondered whether *Tnfrsf13b* governs processes besides production of IgG that could explain the differential aggressiveness of immune responses. To address that question, we surveyed gene expression in GC B cells isolated from the spleens of mice that had been immunized with allogeneic splenocytes and thymocytes (Supplemental Figure 4, Figure 4A, and Supplemental Table 2). As expected, B cells from the GCs, in which IgG responses to allogeneic cells originate, of WT mice exhibited more pronounced expression of Ig light and heavy chain genes (17 genes in WT vs. *Tnfrsf13b*-KO, *P* = 0.0001; and 12/111 genes in WT vs. A144E/A144E, *P* = 0.0028) and genes governing plasma cell differentiation (17/151 genes in WT vs. *Tnfrsf13b*-KO, *P* = 0.002) than GC B cells from mutant mice (Figure 4B). This confirmed the idea that IgG production and B cell maturation do not underlie the aggressive and potentially injurious humoral responses in mice and humans. What could not be anticipated, however, was that GC B cells from immune WT mice also exhibited greater expression of genes associated with immune regulation. Those included *Forkhead box P3* (*Foxp3*), *Indoleamine 2,3-dioxygenase 1* (*Ido1*), *Interleukin 10* (*Il10*), and genes associated with B regulatory cell functions such as *Cd9 antigen* (*Cd9*), *Cd5 antigen* (*Cd5*), *CD1d1 molecule* (*Cd1d1* and *Cd1d2*), and *Hepatitis A virus cellular receptor 1* (*Havcr1*), which encodes T cell Ig mucin-1 (Tim-1), among others (Figure 4B). It is possible these functions supported a modest prolongation of survival of cardiac allografts in several WT mice and could have significance in clinical transplantation where B cells are implicated in immune regulation and tolerance (29). However, the fast development of antibody-mediated injury to transplants in mutant mice is not likely the direct consequence of defective immune regulation alone since cellular immunity developed as fast in WT as in mutant mice (Figure 1, A–D). Accordingly, we questioned whether the aggressive effector responses in mutant mice could reflect deficiency of natural or more rapidly recruited controls of tissue injury, such as availability of natural antibodies (30).

### Control of complement and tissue injury by natural and elicited antibodies.

**Figure 5:**
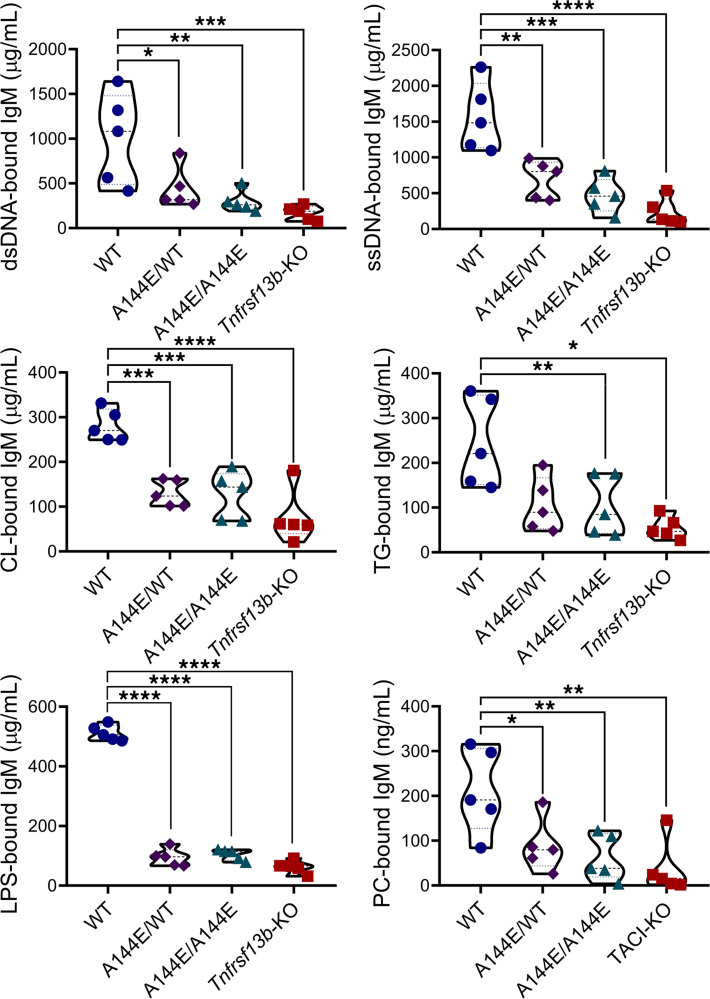
*Tnfrsf13b* controls the production of natural IgM. Mice were immunized via intraperitoneal injection with 5 × 10^7^ BALB/c splenocytes and thymocytes and serum was collected weekly. Serum levels of natural IgM reactive to double-stranded DNA (dsDNA), single-stranded DNA (ssDNA), cardiolipin (CL), thyroglobulin (TG), lipopolysaccharide (LPS), and phosphocholine (PC) 14 days after immunization with allogeneic cells. Graphs are representative of mean ± SEM of 5 mice per group. One-way ANOVA with Dunnett’s multiple comparison test: *P ≤ 0.05; **P ≤ 0.01; ***P ≤ 0.001; ****P ≤ 0.0001 in relation to WT control.

Natural antibodies, so named because they are present without prior exposure to antigen, have been postulated to provide initial defense against pathogens but also to promote repair of cellular injury and control of inflammatory reactions and complement (30–33). We wondered whether a deficiency of natural antibodies might explain the severe manifestations of humoral immunity in *Tnfrsf13b-*mutant mice. *Tnfrsf13b*-deficient, and mono- and biallelic A144E-mutant, mice were indeed deficient of IgM at baseline (Figure 2A and Supplemental Figure 5, A and B) and after immunization with allogeneic cells (Figure 2, A and B; and Supplemental Figure 5, A–C). The deficiency included the fraction of IgM that binds double-stranded DNA (dsDNA), single-stranded DNA (ssDNA), cardiolipin (CL), thyroglobulin (TG), lipopolysaccharide (LPS), and phosphocholine (PC), common targets of polyreactive natural antibodies (Figure 5). Thus, *Tnfrsf13b* controls the amount of IgM “natural” antibodies implicated in the control of inflammation and complement activation.

**Figure 6:**
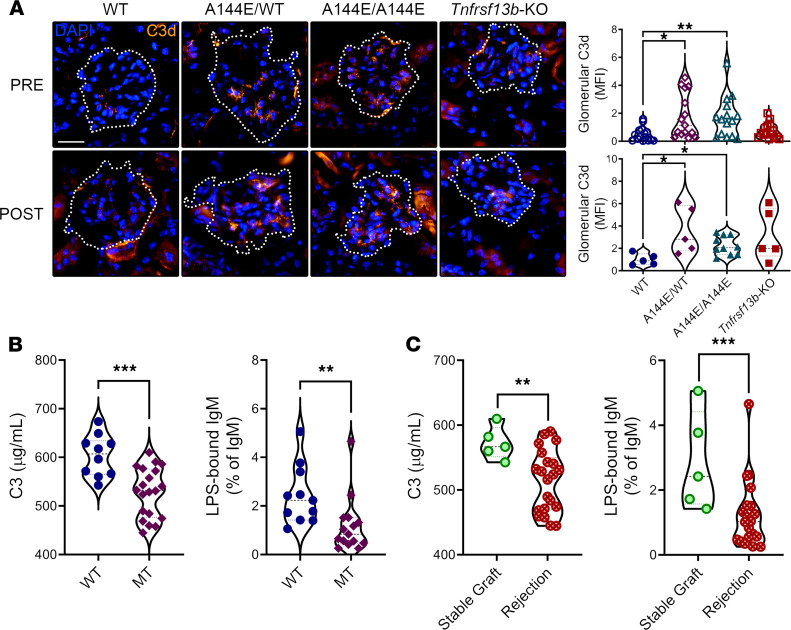
*Tnfrsf13b* controls immune and inflammatory injury. (**A**) Native kidneys were harvested from naive mice (pre) or mice immunized with 5 × 10^7^ BALB/c splenocytes and thymocytes by intraperitoneal injection (post). Glomerular C3d deposits were identified with anti–mouse C3d immunostaining in frozen native kidney sections pre- or 8 days after allogeneic stimulation. The dashed outline represents the glomeruli area as identified with aid of the tissue green autofluorescence and the differential interference contrast in stacked images. Scale bar: 25 μm. Graphs show mean ± SEM calculated from analysis of 5–7 fields with 3 or more glomeruli per mouse per group. Open shapes represent data from naive mice (pre), and filled shapes represent data of mice 8 days postimmunization (post). (**B** and **C**) Blood samples were collected from patients who underwent kidney transplantation. The patients’ *TNFRSF13B* gene exons were sequenced, and the patients were classified according to the absence or presence of missense mutations, as *TNFRSF13B* WT or mutant (MT), respectively, independently of transplantation outcome (**B**) or according to the transplant outcome (**C**). The concentrations of natural LPS-binding IgM and C3 in sera were measured by ELISA. Graphs show mean ± SEM of 8–14 individuals per group. Kruskal-Wallis with Dunn’s multiple comparison test (**A**), Mann-Whitney test (**B** and **C**): *P ≤ 0.05; **P ≤ 0.01; ***P ≤ 0.001; in relation to WT control.

Natural antibodies, especially IgM, regulate complement by reacting with C3b before it can fix on eukaryotic cellular targets (30–32, 34). We therefore reasoned that if the aggressive humoral effector activity in *Tnfrsf13b*-deficient mice owed to deficiency of complement-regulating IgM, excess C3b generated in allografts or by spontaneous alternative pathway tick over (35) would be available to fix to autologous (nontransplanted) tissues. To test that possibility, we examined native kidney tissues from unmanipulated mice and from mice immunized 8 days earlier with allogeneic cells. As Figure 6A shows, the kidneys of unmanipulated mutant mice contained appreciable deposits of C3d, and the deposits were increased with immunization. In contrast, the kidneys of unmanipulated and immunized WT mice contained few or no deposits of C3d. Complement deposition was accompanied by IgM and IgG deposition indicating damage in the native kidney (Supplemental Figure 6).

To test if deficiency of “natural” IgM alone (and not any other feature associated with TACI function) caused complement dysregulation, we measured C3d deposits in the native kidneys in mice that lack “natural” antibodies, the quasi-monoclonal (QM) mouse (36, 37) expressing WT or *Tnfrsf13b-*KO alleles. The QM mouse produces mostly monoclonal IgM that recognizes a synthetic hapten and is not cross-reactive. Immunofluorescence staining of kidney sections obtained from naive QM *Tnfrsf13b-*WT or QM *Tnfrsf13b-*KO mice showed abundant deposition of C3d and IgG (Supplemental Figure 7, A and B). In the absence of “natural” IgM, C3d deposition occurs independently of *Tnfrsf13b* expression, and fully allogenic grafts (BALB/c; H-2^d/d^) were rejected in QM recipients with kinetics comparable to rejection of allografts in C57BL/6 controls (Supplemental Figure 7C). These results suggest that *Tnfrsf13b* promotes production of natural antibodies that regulate complement and possibly other elements of inflammation at baseline and during immune responses to avert incidental injury. In contrast, *Tnfrsf13b* mutations that commonly disrupt the function of the protein leave humoral immunity untethered, allowing increasing injury to foreign targets (and hence host defense) but potentially allowing incidental injury to other tissues and organs.

### TNFRSF13B missense mutations in recipients of kidney transplants are associated with decreased C3 and natural antibodies in the blood.

To glimpse the possibility that defective control of complement in *Tnfrsf13b*-mutant mice might also occur in humans, we measured LPS-binding IgM natural antibodies and C3 in the blood of kidney transplant recipients. As Figure 6B shows, transplant recipients with *TNFRSF13B* missense mutations had significantly lower concentrations of LPS-bound IgM natural antibodies as a fraction of IgM, and less C3 in serum than transplant recipients with 2 WT alleles, independent of transplant outcome. Reduced C3 levels and LPS-bound IgM in the blood after transplantation were also associated with AMR as compared with the group with a good outcome (Figure 6C). These findings suggest that *TNFRSF13B* missense mutations decrease natural antibodies and increase C3 activation.

## Discussion

Here we show that *TNFRSF13B* controls the character and aggressiveness of immune responses. In contrast to MHC, *TNFRSF13B* did not affect the initiation of an immune response per se but rather its regulation by virtue of controlling “natural” antibodies produced by innate B cells. Thus, by inhibiting natural antibody secretion (IgM and IgA) and favoring adaptive antibody responses, *TNFRSF13B* diversity establishes varying susceptibility to infection (9, 38), autoimmunity (39, 40), immunodeficiency (15, 21, 41–43), and excessive inflammatory reactions to transplantation and perhaps to pathogens as well.

The *TNFRSF13B* deficiency heightened propensity for AMR, and particularly increased deposition of C3d, results from 2 related features of the mutant phenotype. First, *Tnfrsf13b* mutants have lower baseline levels of Ig and notable deficiency of IgM and IgA (28, 42) (produced by marginal zone B cells and peritoneal B1 cells); but mutants can still produce high-affinity IgG (in GCs) (9). The high-affinity IgG produced by mutant mice effectively prevents certain infections (9), and here we show it can initiate AMR. But as important as production of high-affinity IgG in contributing to rejection may be the second feature of the phenotype, which is the scarcity of IgM and IgA. All Ig, but especially IgM and IgA, can provide alternative substrate for fixation of C3b. If Ig binds weakly or not at all to a target, the fixation of C3b diverts this active moiety away from cell surfaces, effectively decreasing local complement fixation (35). Tightly bound Ig in contrast has the opposite effect, potentially increasing the amount of local complement activation. Thus, mutant mice have the ability to produce allospecific antibodies but lack the regulatory activity conferred by unbound and weakly bound Ig.

The physiological impact of some *TNFRSF13B* polymorphisms has been determined (44–46) and simultaneously manifests benefits and risks evidenced by balancing selection at this locus (12), but the relationship between many polymorphisms and specific phenotypes remains to be fully elucidated (10). *TNFRSF13B* genotypes that disrupt the function of the encoded protein favor highly aggressive humoral immune responses. The WT allows both cell-mediated and antibody-mediated responses to proceed, albeit with restraint. Although we used immune responses to transplantation in mice and humans to explore the impact of disruptive genes on the phenotype (since every animal and human potentially mounts a biologically impactful response), it is not difficult to imagine how the range of phenotypes of *TNFRSF13B* variants would affect host defense and disease. For example, *Tnfrsf13b*-mutant mice mount faster and more effective B cell responses than WT mice to enteric pathogens that model enteropathogenic *E*. *coli* that causes widespread acute and chronic enteritis (9). We recently found that mutant strains with *Tnfrsf13b* haploinsufficiency or deficiency (38) also delimit transmission (47), and that might explain the apparently positive selection of disabling variants (11–13). Yet, the WT *TNFRSF13B* and *Tnfrsf13b* also have favorable characteristics. Not only does the WT allow full maturation of B cell responses (28), but we also show here that the WT also governs the extent of inflammation and activation of complement that occur at baseline and upon exposure to antigen by controlling the functions of innate B cells. The importance of control of inflammation and complement reactions has been recently highlighted by untoward responses in some infected with SARS-CoV-2 (48, 49).

The profound diversity of *TNFRSF13B* and *Tnfrsf13b* across mammalian species (11) further supports the concept that phenotypes we describe in transplant recipients have broader biological significance. The limited analysis of *TNFRSF13B* sequences reveals as much diversity as MHC and suggests that both the WT and missense variants are sustained by positive selection even though most individual SNPs are rare and do not perturb expression of the protein (11–13). Although the full extent of *TNFRSF13B* diversity and implications for physiology remain to be established (doing so will depend on meticulous sequencing of all exons and regulatory sequences), our results suggest the potential value. The need for full sequencing of polymorphic genes is highlighted by the failure of genome-wide association studies to identify *TNFRSF13B* SNPs as a risk factor for rejection (50–52).

In transplantation much effort has been devoted to identifying recipients at heightened risk of developing AMR because this type of rejection poses the most significant risk for early demise of organ grafts. Sensitization to donor antigens poses such a risk that transplantation might be delayed or avoided entirely if it is detected. However, because most transplant recipients are not presensitized, the risk of antibody-mediated disease is uncertain. Probing *TNFRSF13B* sequence and/or function might address that question and help explain why some individuals with autoantibodies develop autoimmune disease while many with the same antibodies have no evident disease and why some individuals mount destructive inflammatory responses following viral infections while others remain asymptomatic. Since the receptor encoded by *TNFRSF13B* is actionable by modified ligands and/or antibodies, our discovery suggests a new avenue for antiinflammatory therapeutics, either supplementing intermediates produced by *TNFRSF13B* or providing the appropriate end products for those lacking or having decreased *TNFRSF13B* function.

## Methods

### Human participants.

The experimental cohorts were drawn from the active and inactive patients in the transplant nephrology service at the University of Michigan. The cohorts were matched for donor variables (age, race, sex, height, weight, creatinine, diabetes, hypertension, cigarette use, hepatitis C); transplant variables of pulsatile perfusion, cold ischemia time, organ sharing (local, regional, national), HLA mismatch score, year of transplant, en bloc/double, and ABO compatibility; and recipient variables of age, race, sex, diagnosis, pretransplant blood transfusion, body mass index, peak panel reactive antibodies (PRA)/calculated PRA, pretransplant years of dialysis, immune-suppressive therapy including use of T cell depletion agents, angina, peripheral vascular disease, drug-treated chronic obstructive pulmonary disease, and hepatitis C virus infection. The patients were identified using the list of transplant recipients currently or previously followed at the University of Michigan or at the University of Wisconsin, curated by the Organ Transplantation Information System and in coordination with the honest broker’s office at the University of Michigan. The presence or absence of rejection was defined based on decreased graft function and biopsy findings typical of AMR. Diagnosis of AMR is usually based on pathological findings that include C4d deposition in peritubular capillaries or glomerular capillaries, peritubular or glomerular capillary inflammation, and glomerular basement membrane duplication. In the AMR group we also included patients with donor-specific antibodies that were persistent, i.e., found in more than 1 occasion, even if there was no evidence of rejection on the biopsy. Protocol biopsies were examined for presence of C4d and/or glomerulitis and/or peritubular capillaritis (53). Stable graft function was defined as absence of an unexplained decrease in the estimated glomerular filtration rate (eGFR) more than 15% from baseline. Unexplained decreases in the eGFR usually prompt a transplant biopsy and clinical testing of donor-specific antibodies. Individuals with proteinuria more than 500 mg were excluded to avoid uncertainties about etiology (i.e., transplant glomerulopathy, which rarely causes significant proteinuria in the first year, versus de novo or recurrent disease).

### TNFRSF13B sequencing and analysis.

The *TNFRSF13B* gene is located on chromosome 17 and is composed of 5 exons spanning 34 kb. We amplified exons 1, 2, 3, and 4 with the primers Salzer and colleagues described (41). Exon 5 was amplified using 2 pairs of primers: 5′-CTGCCCACACCGTCACCCCTACC-3′ and 5′-CTCTCCCCTCTCCCCACCTCTC-3′, and 5′-GGGGGTCAGGGAGGGAAAGGAG-3′ and 5′-TGATGCCCAGGAAAGTGATAGACAAG-3′. Genomic DNA was extracted from peripheral blood cells using DNeasy Blood & Tissue Kit following the manufacturer directions (QIAGEN 69504). Alternatively, DNA samples were obtained from the Michigan Genome Initiative. PCR was performed with Taq DNA Polymerase, native (Thermo Fisher Scientific 18038-042), at 95°C for 15 minutes, 46 cycles of 94°C for 30 seconds + 60°C for 15 seconds + 72°C for 30 seconds, followed by 72°C for 5 minutes. Samples were run in a 2% agarose gel; specific bands were cut out, and the DNA was purified with QIAquick Gel Extraction Kit (QIAGEN 28706). Sequences were obtained by Sanger sequencing performed at the University of Michigan Sequencing Core facility and aligned using Sequencher 5.4.6 software (Gene Codes Corporation).

### Mice.

BALB/cJ (000651), CB6F1/J (100007), and C57BL/6J (000664) *Tnfrsf13b*-WT mice were purchased from The Jackson Laboratory. *Tnfrsf13b*-KO mice (54) and mice harboring biallelic (A144E/A144E) or monoallelic A144E mutations (A144E/WT) (38), homologous to the human A181E mutation, were previously described. QM mice have been described previously (36, 37) and were bred with *Tnfrsf13b*-KO mice to produce QM *Tnfrsf13b*-KO mice. All the *Tnfrsf13b*-mutant mice were bred onto the C57BL/6 background. Animal experimentation was performed in mice of both sexes between 8 and 20 weeks of age.

### Heart and skin allografts.

Hearts from CB6F1 (C57BL/6-BALB/J F1, H-2^b/d^ haplotype) mice were transplanted heterotopically into the abdomen of C57BL/6 background WT and *Tnfrsf13b*-deficient mice (H-2^b/b^ haplotype). Hybrid mice sharing a haplotype with the recipient were used as sources of grafts to minimize the impact of natural killer cells. Alternatively, hearts from BALB/c (H-2^d/d^) were transplanted heterotopically into the abdomen of C57BL/6 background QM and QM *Tnfrsf13b*-deficient mice (H-2^b/b^ haplotype). Heterotopic cardiac transplants do not confer cardiac function in the recipient because the heart muscle is perfused. Heart chambers are not connected to the circulation, but the grafts can undergo rejection as would an experimental or clinical transplant. The grafts were palpated daily, and rejection was considered when the hearts stopped beating. The mice were sacrificed on rejection day, and the transplanted hearts, spleens, and sera were collected. All the transplanted mice were maintained in the absence of immunosuppression. Skin grafts from CB6F1 were transplanted into C57BL/6 background WT and *Tnfrsf13b*-deficient mice. The grafts were observed daily, and rejection was considered when 50% of the graft became necrotic. All the transplanted mice were maintained in the absence of immunosuppression.

### Allogeneic stimulation.

WT, *Tnfrsf13b*-deficient, and biallelic and monoallelic *Tnfrsf13b* A144E-mutant mice (H-2^b/b^ haplotype) were immunized by intraperitoneal injection with 5 × 10^7^ BALB/c splenocytes and thymocytes (H-2^d/d^ haplotype). After 8–21 days the mice were sacrificed, and the sera, spleens, and kidneys were collected for posterior analysis. Alternatively, nonimmunized mice were used as controls.

### Flow cytometry and antibodies.

Splenocytes and peritoneal cells were isolated from mice immunized with allogeneic cells, from transplanted mice, or from naive mice, then counted and frozen for posterior analysis. The cell viability was assessed by staining with BD Horizon Fixable Viability Stain 780 (FVS780; 1.11 μg/mL; BD Biosciences 565388). Splenocytes were stained with FITC-conjugated antibodies rat anti–mouse CD19 (1D3; 10 μg/mL; BD Biosciences 553785, RRID:AB_395049), CD23 (B3B4; 10 μg/mL; BD Biosciences 553138, RRID:AB_394653), or Armenian hamster anti–mouse CD95 (Jo2; 10 μg/mL; BD Biosciences 554257, RRID:AB_395329); PE-conjugated rat anti–mouse CD19 (1D3; 4 μg/mL; BD Biosciences 557399, RRID:AB_396682), GL7 (GL7; 4 μg/mL; BD Biosciences 561530, RRID:AB_10715834), or IgD (11-26c.2a; 4 μg/mL; BD Biosciences 558597, RRID:AB_647211); PerCP-Cy5.5–conjugated rat anti–mouse CD21/CD35 (7G6; 4 μg/mL; BD Biosciences 562797, RRID:AB_2737802); and APC-conjugated rat anti–mouse B220 (RA3-6B2; 4 μg/mL; Thermo Fisher Scientific 17-0452-81, RRID:AB_469394) or CD19 (1D3; 4 μg/mL; BD Biosciences 550992, RRID:AB_39848). Multiparametric flow cytometric analysis of 10^6^ stained cells was performed using a BD FACSCanto II (BD Biosciences), and 100,000 events were analyzed with FlowJo 10 software (FlowJo, LLC, RRID:SCR_008520). In addition, splenocytes and peritoneal cells from mice immunized with allogeneic cells for 10 days were stained with, respectively, FVS780, anti-CD19 APC, GL7 PE, and CD95 FITC; and FVS780, anti-CD19 PerCP-Cy5.5, CD23 PE-Cy7, IgM Alexa Fluor 488, and IgD PE as described above. GC B cells (FVS780^–^CD19^+^GL7^+^CD95^+^) (Supplemental Figure 4A) were sorted in a Synergy SY3200 Cell Sorter (Sony Biotechnology) at the University of Michigan Flow Cytometry Core.

### RNA extraction and gene expression analysis of GC B cells.

The RNA from the sorted GC B cells was extracted with the RNeasy Plus Mini Kit (QIAGEN 74134) as recommended by the manufacturer. Next, the RNA integrity number (RIN) and amount were analyzed by RNA quality control analysis using Agilent RNA 6000 Pico Kit (Agilent Technologies 5067-1513) in an Agilent 2100 Bioanalyzer (Agilent Technologies). All the samples used for the microarray analysis had RIN values between 8.8 and 10. The gene expression was assessed using a GeneChip Mouse Gene 2.1 ST Array Plate (Affymetrix 902140). The distribution of the probes for each chip was analyzed (Supplemental Figure 4B), and the standard error for each gene on each array was assessed after fitting a probe-level model (Supplemental Figure 4C). Expression values for each gene were calculated using a robust multiarray average (55) and transformed in log_2_ values. Values were fit in a principal component analysis, and the first 2 principal components were plotted to show sample gene expression clusters (Supplemental Figure 4D). Data were fit to weighted linear models (56), and the contrasts of interest were computed. Additionally, the expression data were weighted in a gene-by-gene update algorithm designed to downweight chips that are deemed less reproducible (57). The probe sets with a fold change greater than 2 were selected, and the *P* values were adjusted for multiple comparison using false discovery rate (58). A *P* value equal or less than 0.05 was deemed significant. All analyses were performed using oligo and limma packages of bioconductor implemented in the R statistical environment (R version 3.4.3). Additionally, the gene expression data were loaded into iPathwayGuide software (Advaita Bioinformatics), and cellular process pathways were analyzed. Data were deposited in NCBI Gene Expression Omnibus under accession number GSE182739.

### Enzyme-linked ImmunoSpot for detection of immunoglobulin-secreting cells.

Filtration Plate MultiScreen HTS HA Sterile Plates (96 wells, MilliporeSigma MSHAS4510) were activated and coated for 1 hour at room temperature with goat anti–mouse Ig (H+L) (4 μg/mL; SouthernBiotech 1010-01, RRID:AB_2794121). After blocking overnight at 4°C, mouse splenocytes or peritoneal cells were plated in serial dilutions, starting with 10^5^ cells per well, and incubated at 37°C in 5% CO_2_ atmosphere overnight. Spots of bound IgG or IgM were detected by adding the alkaline phosphatase–conjugated antibodies goat anti–mouse IgG (0.5 μg/mL; SouthernBiotech 1030-04, RRID:AB_2794293) or goat anti–mouse IgM (0.5 μg/mL; SouthernBiotech 1020-04, RRID:AB_2794200) for 1 hour at room temperature. The reaction was visualized by subsequent addition of 5-bromo-4-chloro-3-indolylphosphate/nitro blue tetrazolium substrate (MilliporeSigma B5655-5TAB). The number of spots was assessed via a CTL ImmunoSpot S5 UV Analyzer equipped with ImmunoSpot ImmunoCapture and ImmunoSpot Counting softwares (Cellular Technology Ltd., RRID:SCR_011082).

### ELISA for detection of mouse immunoglobulins.

Nunc MaxiSorp ELISA plates (Thermo Fisher Scientific 44-2404-21) were coated overnight with goat anti–mouse Ig (H+L) (4 μg/mL; SouthernBiotech 1010-01, RRID:AB_2794121). After blocking, the plates were incubated with transplanted or allogeneically stimulated mice serum. Bound IgG or IgM was detected by adding goat anti–mouse IgG–HRP (4 μg/mL; SouthernBiotech 1030-05, RRID:AB_2619742) or goat anti–mouse IgM–HRP (4 μg/mL; SouthernBiotech 1020-05, RRID:AB_2794201). Alternatively, polyreactive natural IgM was detected by analyzing the amount of immunoglobulin bound to LPS, TG, CL, dsDNA, ssDNA, or PC adapted from the protocol Singh et al. described (59). Briefly, Nunc MaxiSorp ELISA plates were coated overnight at room temperature with TG (10 μg/mL; Alpha Diagnostic International THGL15-N-1), CL (10 μg/mL; MilliporeSigma C0563-10MG), LPS (10 μg/mL; Alpha Diagnostic International LPS12-1), dsDNA, ssDNA (10 μg/mL; MilliporeSigma D8515-1G), or PC (10 μg/mL; MilliporeSigma P0378). After blocking, mice sera were incubated for 2 hours at 37°C. Bound IgM was detected by adding goat anti–mouse IgM–HRP (4 μg/mL; SouthernBiotech 1020-05; RRID:AB_2794201). The reactions were visualized by subsequent addition of 2,2′-Azino-bis (3-ethylbenzthiazoline-6-sulfonic acid) substrate (SouthernBiotech 0202-01). All readings were recorded at 405 nm.

### Allospecific antibodies’ detection by flow cytometry.

Titers of allospecific IgG and IgM in the sera before and after the heart transplant rejection and at different times after the immunization with allogeneic cells were assessed by flow cytometry. Briefly, BALB/c thymocytes (allogeneic) were incubated with different concentrations of serum for 30 minutes at 4°C. The thymocytes were washed and bound antibodies were detected with Cy5-conjugated goat anti–mouse IgG (4 μg/mL, SouthernBiotech 1030-15, RRID:AB_2794299) or Alexa Fluor 488–conjugated goat anti–mouse IgM (4 μg/mL, SouthernBiotech 1020-30, RRID:AB_2794219) for 30 minutes at 4°C and analyzed in a BD FACSCanto II (BD Biosciences). The MFIs in the APC channel (measuring bound IgG) and FITC channel (measuring bound IgM) were determined with FlowJo 10 software (FlowJo, LLC, RRID:SCR_008520) inside the lymphocyte gate.

### Histopathology and immunofluorescence.

Upon sacrifice, the transplanted hearts were sectioned, and part was fixed, embedded in paraffin, and stained with H&E to perform the histological studies. Alternatively, sections of the transplanted heart and naive and immunized mouse kidneys were snap-frozen in Tissue-Tek Optimal Cutting Temperature Compound (Sakura Finetek 4583) and snap-frozen. Five-micron cryosections were processed, and the native and transplanted heart sections were incubated with the primary antibodies goat anti–mouse C3d (800 ng/mL, R&D Systems AF2655, RRID:AB_2066622), goat anti–mouse IgM, human adsorbed (10 μg/mL; Southern Biotech 1020-01, RRID:AB_2794197), for 1 hour at 4°C, followed by CF555-conjugated donkey anti–goat IgG (20 μg/mL; MilliporeSigma SAB4600059-250UL) or Alexa Fluor Plus 488–conjugated donkey anti–goat IgG (4 μg/mL; Thermo Fisher Scientific A32814, RRID:AB_2762838); and rat anti–mouse CD4 (GK1.5) (10 μg/mL; eBioscience 14-0041-86, RRID:AB_467065) or rat anti–mouse CD8a (53-6.7) (10 μg/mL; BD Biosciences 553027, RRID:AB_394565) for 1 hour at 4°C, followed by CF488-conjugated goat anti–rat IgG (20 μg/mL; MilliporeSigma SAB4600046-250UL); rabbit anti–mouse FoxP3 (2 μg/mL; Novus Biologicals NB100-39002SS, RRID:AB_1290944) for 1 hour at 4°C, followed by CF555-conjugated goat anti–rabbit IgG (20 μg/mL; MilliporeSigma SAB4600068-250UL, RRID:AB_2336059); or Texas Red-X–conjugated goat anti–mouse IgG (8 μg/mL; Thermo Fisher Scientific T-862, RRID:AB_2556781) incubated for 1 hour at 4°C. Additionally, native kidneys from naive mice and mice immunized with allogeneic cells were stained with anti-IgM, anti-IgG, and anti-C3d as described above. For contrast, nuclei were stained with DAPI, and slides were mounted with antifade mounting media. The slides were examined with a Leica DMI6000B microscope equipped with a Leica DFC360 FX monochrome digital camera and a Leica HCX PL FLUOTAR L 40X/0.60 CORR PH2 microscope objective (Leica Microsystems). At least 5 fields were imaged at 400× original magnification via QCapture Pro 7 software (QImaging, RRID:SCR_014432). For the native kidney slides, the glomeruli area was selected with aid of the tissue green autofluorescence and the differential interference contrast in stacked images, and the MFI of IgM, IgG, or C3d in the selected area was assesed via Adobe Photoshop CC software (Adobe Systems Inc., RRID:SCR_014199).

### ELISA for detection of human immunoglobulins.

Nunc MaxiSorp ELISA plates (Thermo Fisher Scientific 44-2404-21) were coated overnight with goat anti–human IgM (4 μg/mL; SouthernBiotech 2020-01, RRID:AB_2795599). After blocking, the plates were incubated with serum from transplanted patients. Bound IgM was detected by adding HRP-conjugated goat anti–human IgM (4 μg/mL; SouthernBiotech 2020-05, RRID:AB_2795603). Alternatively, polyreactive natural IgM was detected by analyzing the amount of immunoglobulin bound to LPS adapted from the protocol Singh et al. described (59). Briefly, Nunc MaxiSorp ELISA plates were coated overnight at room temperature with LPS (10 μg/mL; Alpha Diagnostic International LPS12-1). After blocking, human sera were incubated for 2 hours at 37°C. Bound IgM was detected by adding goat anti–human IgM–HRP (4 μg/mL; SouthernBiotech 2020-05, RRID:AB_2795603). The reactions were visualized by subsequent addition of 2,2′-Azino-bis (3-ethylbenzthiazoline-6-sulfonic acid) substrate (SouthernBiotech 0202-01). All readings were recorded at 405 nm.

### ELISA for detection of human C3.

The concentration of complement component 3 in the serum of the kidney transplant recipients was assessed with the Human Complement C3 ELISA Kit (Abcam ab108822) according to the manufacturer’s instructions. Reference values: 800 to 1600 μg/mL.

### Statistics.

All comparisons were done with GraphPad Prism 8 software (GraphPad Software, RRID:SCR_002798). A *P* value of equal or less than 0.05 was considered significant. Detailed statistics for each panel can be found in the tables and figures’ legends. To determine if any observed alleles were associated with the development of AMR, we compared the number of mutated and WT alleles and patients harboring the mutations in rejecting individuals and in control samples using Fisher’s exact test. χ^2^ test was used to verify if there was a relationship between the *Tnfrsf13b* genotype and early (up to 3 weeks) cardiac allograft rejection or skin graft rejection (2 weeks).

### Study approval.

Written informed consent was obtained from all human participants prior to inclusion in the study. Research was approved by the Institutional Review Board of the University of Michigan Medical School. Mice were maintained under specific pathogen–free conditions, and all the experiments were performed in accordance with the approved animal protocols and the regulations of University of Michigan Committee on the Use and Care of Animals.

## Author contributions

MC and JLP conceived the study; MGMB, MC, and JLP designed the methodology; MGMB, ARL, DH, HL, YZ, BF, JB, and EAF performed experiments; MGMB and MC wrote the original draft; MGMB, EAF, MS, RJB, RSG, AS, ETLP, JLP, and MC reviewed and edited the draft; MGMB, MC, JLP, and AS acquired funding; MGMB, MC, JLP, and EAF provided resources; and MGMB, MC, and JLP provided supervision.

## Supplementary Material

Supplemental data

Supplemental table 2

## Figures and Tables

**Table 1:**
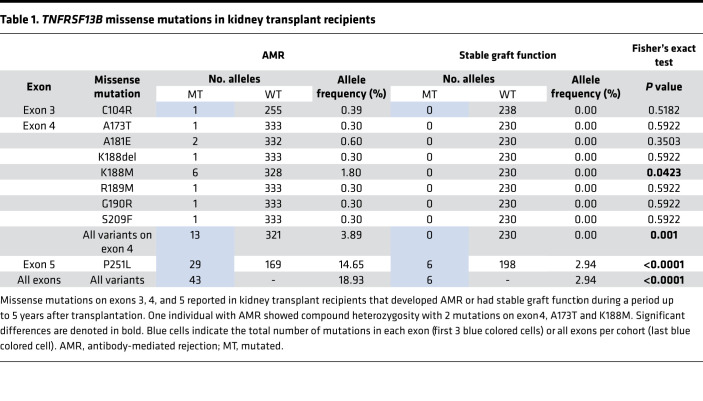
*TNFRSF13B* missense mutations in kidney transplant recipients

**Table 2:**
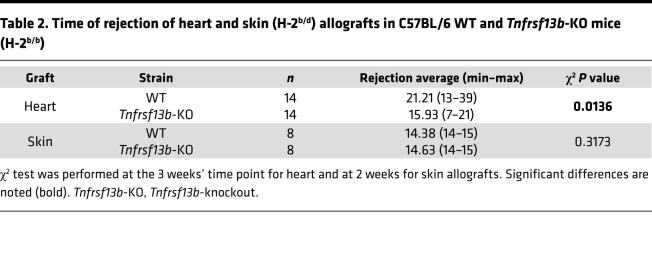
Time of rejection of heart and skin (H-2^b/d^) allografts in C57BL/6 WT and *Tnfrsf13b*-KO mice (H-2^b/b^)

## Version 1 07/20/2021

In-Press Preview

## Version 2 09/08/2021

Electronic publication
