# Supplementary material for: TNFRSF13B genotypes control immune-mediated pathology by regulating the functions of innate B cells
Source: JCI Insight. 2021 Sep 8;6(17):e150483. doi: 10.1172/jci.insight.150483 (PMC8492324; doi:10.1172/jci.insight.150483)
Supplement: Supplemental data [file jciinsight-6-150483-s303.pdf]

Title: ***TNFRSF13B* genotypes control immune-mediated pathology by regulating the functions of innate B cells**

Authors: Mayara Garcia de Mattos Barbosa[1], Adam R. Lefferts[1], Daniel Huynh[1], Hui Liu[1], Yu Zhang[1], Beverly Fu[1], Jenna Barnes[2], Milagros Samaniego[3], Richard J. Bram[4], Raif S. Geha[5], Ariella Shikanov[6], Eline T. Luning Prak[7], Evan Farkash[2], Jeffrey L. Platt[1,8,*], Marilia Cascalho[1,8,*]

[1]Department of Surgery, University of Michigan, Ann Arbor, MI, 48109, USA

[2]Department of Pathology, University of Michigan, Ann Arbor, MI, 48109, USA

[3]Department of Medicine, University of Michigan, Ann Arbor, MI, 48109, USA

[4]Department of Pediatric and Adolescent Medicine and Department of Immunology; Mayo Foundation; Rochester, MN, 55905, USA

[5]Division of Immunology, Boston Children's Hospital and Department of Pediatrics; Harvard Medical School; Boston, MA, 02115, USA

[6]Department of Biomedical Engineering; University of Michigan; Ann Arbor, MI, 48109, USA

[7]Department of Pathology and Laboratory Medicine; Perelman School of Medicine; University of Pennsylvania; Philadelphia, PA, 19104, USA

[8]Department of Microbiology and Immunology; University of Michigan; Ann Arbor, MI, 48109, USA

Supplemental data and figures:

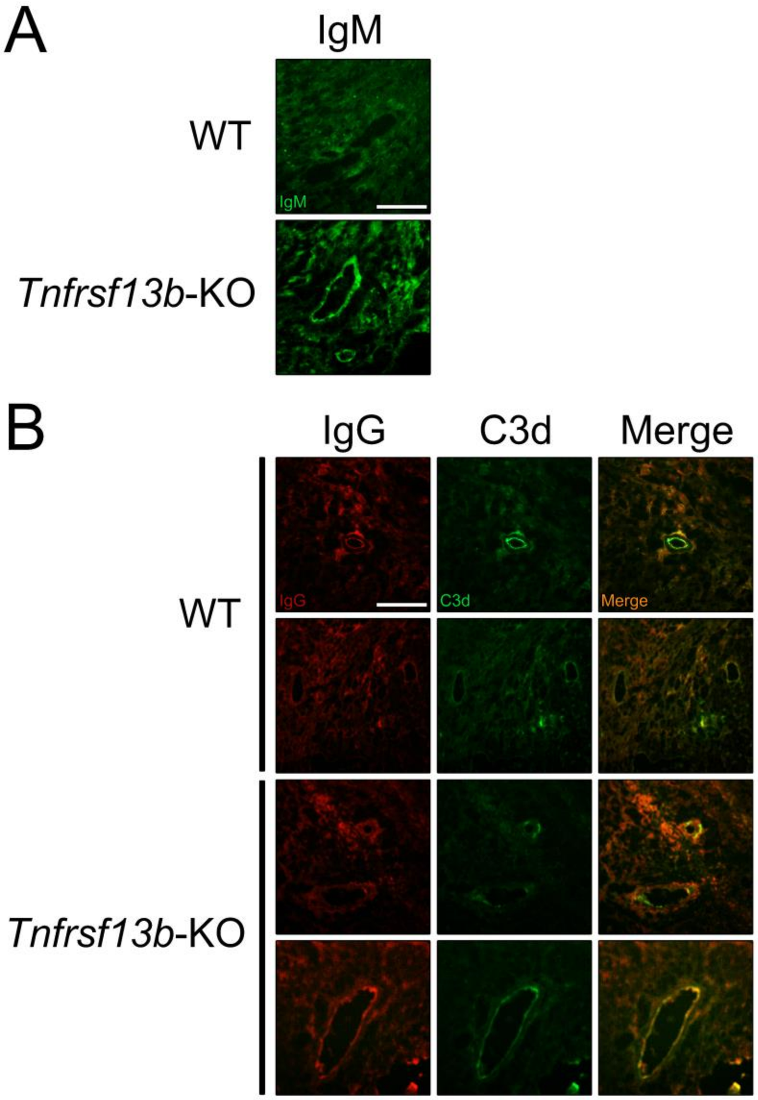

Figure S1. Anti-IgM, IgG and C3d immunostainings in transplanted hearts 14 days after transplantation. Hearts from CB6F1 mice (C57BL/6-BALB/c F1, H-2$^{b/d}$ haplotype) were transplanted heterotopically into the abdomen of C57BL/6 (H-2$^{b/b}$ haplotype) WT and *Tnfrsf13b*-KO. Deposition of IgM, IgG and C3d was evaluated by immunofluorescence. **(A)** Anti-IgM immunostaining of sections obtained from cardiac allografts excised at day 14. Images are representative of a mouse of each group. **(B)** Anti-IgG and anti-C3d immunostainings of sections obtained from cardiac allografts at rejection. Images are representative of two mice of each group. See also Figure 1. Bars: 40 µm.

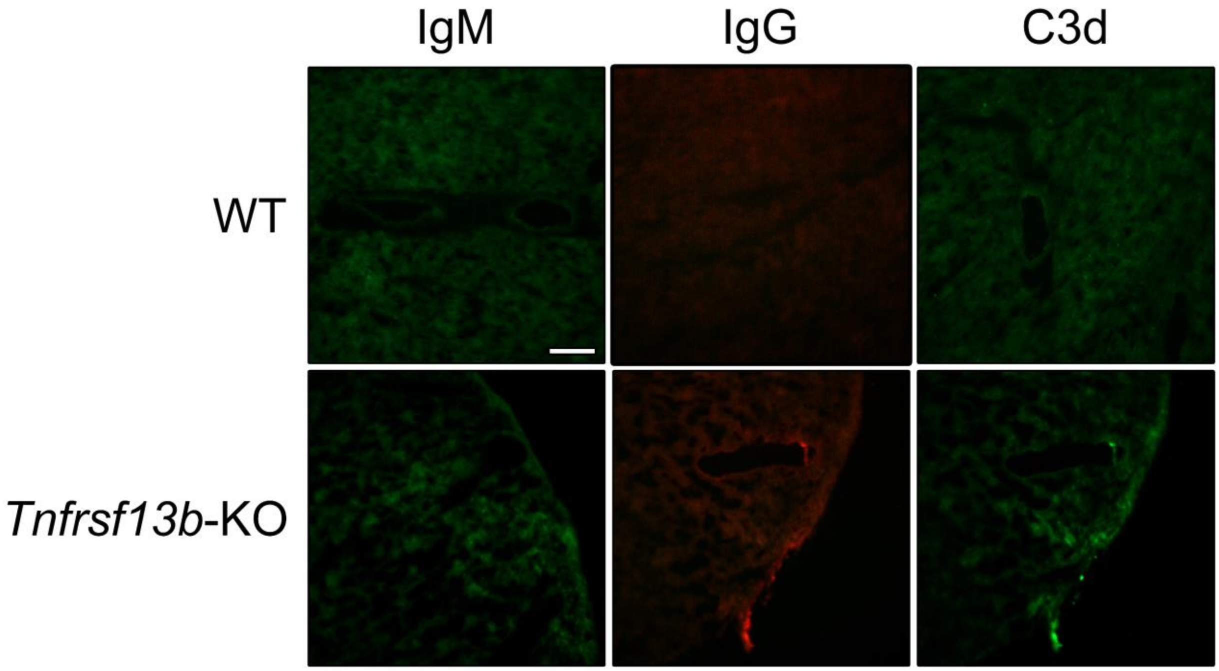

Figure S2. Anti-IgM, IgG and C3d immunostainings of sections of native hearts obtained at rejection of cardiac allografts. Hearts from CB6F1 mice (C57BL/6-BALB/c F1, H-2$^{b/d}$ haplotype) were transplanted heterotopically into the abdomen of C57BL/6 (H-2$^{b/b}$ haplotype) WT and *Tnfrsf13b*-KO. Both transplanted and native hearts were retrieved at rejection. Deposition of IgM, IgG and C3d into the native heart was evaluated by immunofluorescence. Images are representative of a mouse of each group. Bar: 40 µm.

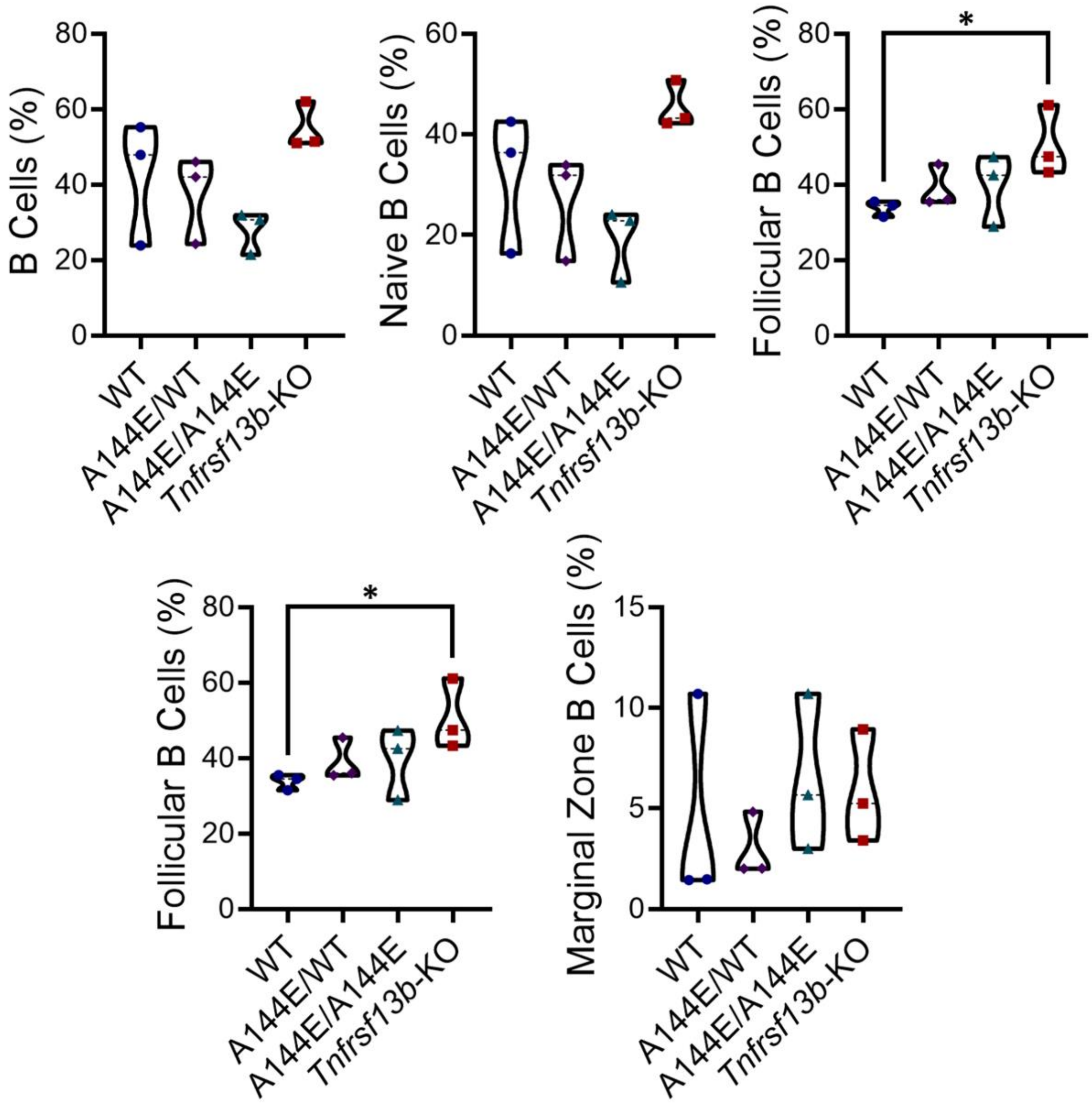

Figure S3. Splenic B cell populations in *Tnfrsf13b*-mutant mice. Spleens from naïve mice with different *Tnfrsf13b* genotypes were harvested. Splenic percentages of live lymphocytes with phenotypes of B cells ($CD19^+$), naive B cells ($CD19^+$ IgD^+) and percentages of $CD19^+$ B cells that were marginal zone ($CD19^+$ $CD21^{high}$ $CD23^-$), follicular ($CD19^+$ $CD21^+$ $CD23^+$) and germinal center ($CD19^+$ $CD95^+$ $GL7^+$) B cells in the spleen. Graphs are representative of Mean ± SEM of 3 naïve mice per group. Mann-Whitney test: * $p \leq 0.05$ in relation to the WT control.

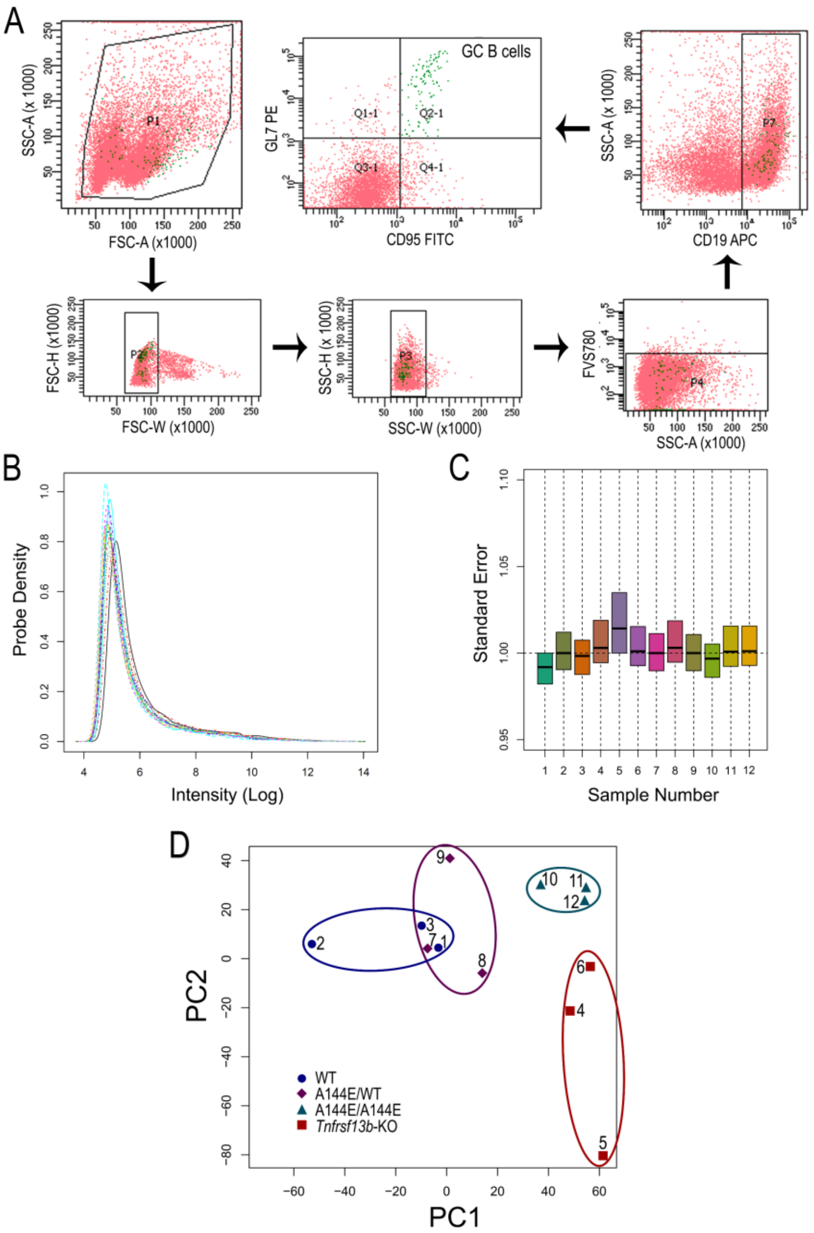

Figure S4. Germinal center B cells sorting and microarray quality analysis. Mice were immunized by intraperitoneal injection with 5 x 10^7 allogenic splenocytes and thymocytes. Spleens were collected after 10 days, germinal center (GC) B cells were sorted, RNA was extracted, and gene expression was analyzed by microarray. **(A)** Singlet viable lymphocytes that were $CD19^+$ (B cells) were further analyzed for the expression of GL7 and FAS (CD95). B cells with a GC phenotype ($CD19^+$ $CD95^+$ $GL7^+$) were sorted (green) for RNA microarray analysis. **(B)** Probe densities in each microarray chip analyzed (n = 3). **(C)** Box plot shows the standard errors for each array calculated after fitting a probe-level model. **(D)** Distribution of analyzed samples in the first two principal components, responsible for 31% of the variation in the principal component analysis. Colored ellipses represent clustered samples by genotype. Sample numbers: 1-3 WT, 4-6 *Tnfrsf13b*-KO, 7-9 A144E/WT, 10-12 A144E/A144E.

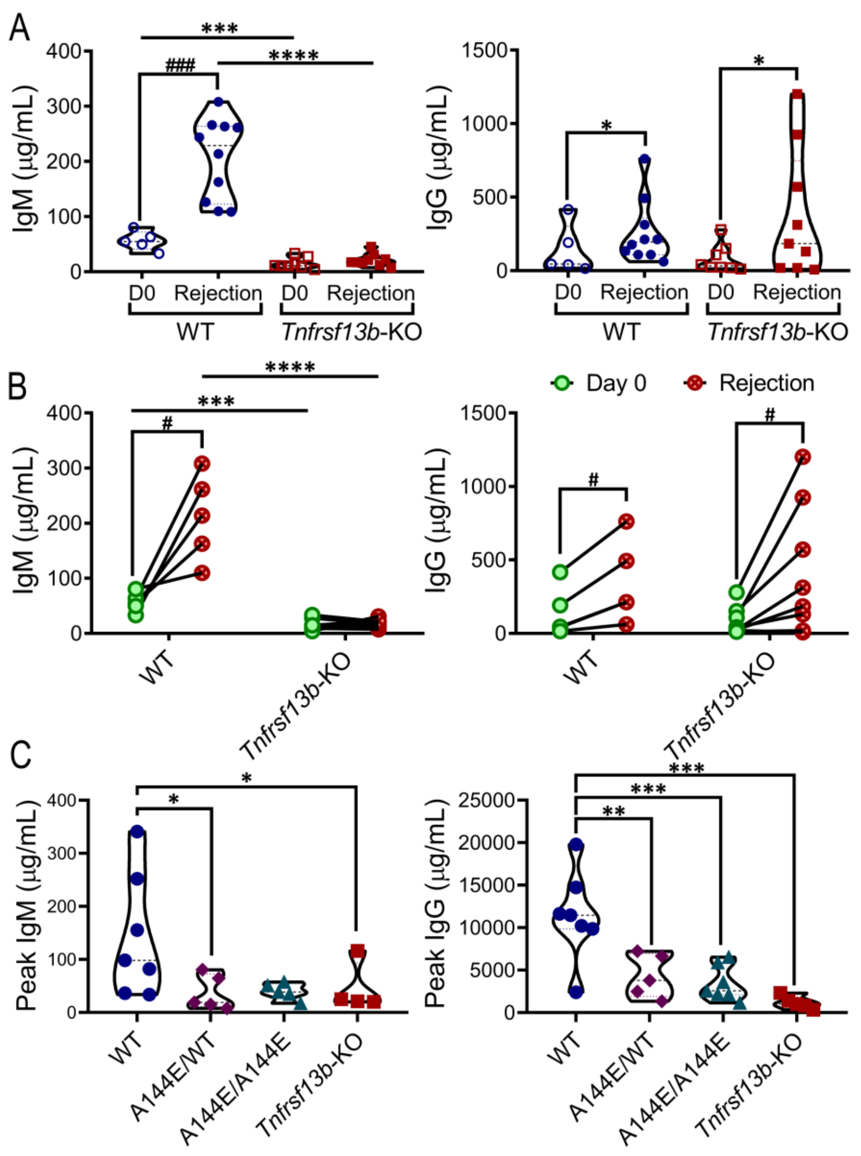

Figure S5. *Tnfrsf13b* deficiency evokes decreased IgM and increased IgG responses to allografts and allo-immunization. (A and B) Hearts from CB6F1 mice (C57BL/6-BALB/c F1, H-2$^{b/d}$ haplotype) were transplanted heterotopically into the abdomen of C57BL/6 (H-2$^{b/b}$ haplotype) WT and *Tnfrsf13b*-KO mice and sera of recipient mice were collected at time of transplant and at rejection. **(A)** Graphs represent the Mean ± SEM of concentrations of total IgM and IgG before transplantation (Day 0) and at rejection of 5-7 mice per group. **(B)** Paired analysis of immunoglobulin concentrations before transplantation (Day 0, green) and at rejection (red). **(C)** Mice were immunized via intraperitoneal injection with 5 x 10^7 BALB/c splenocytes and thymocytes, blood was collected weekly for 21 days. The concentrations of total IgM and IgG at the peak of the response post-immunization with allogeneic cells. Graphs are representative of Mean ± SEM of 5-7 mice per group. Unpaired **(A, B),** paired T test **(B),** Mann-Whitney test **(B, C),** One-Way ANOVA with Dunnett's multiple comparison test or Kruskal-Wallis with Dunn's multiple comparison test **(C)**: * $p \leq 0.05$; ** $p \leq 0.01$; *** $p \leq 0.001$; **** $p \leq 0.0001$ in relation to WT control; # $p \leq 0.05$; ### $p \leq 0.001$ in relation to day 0 (D0).

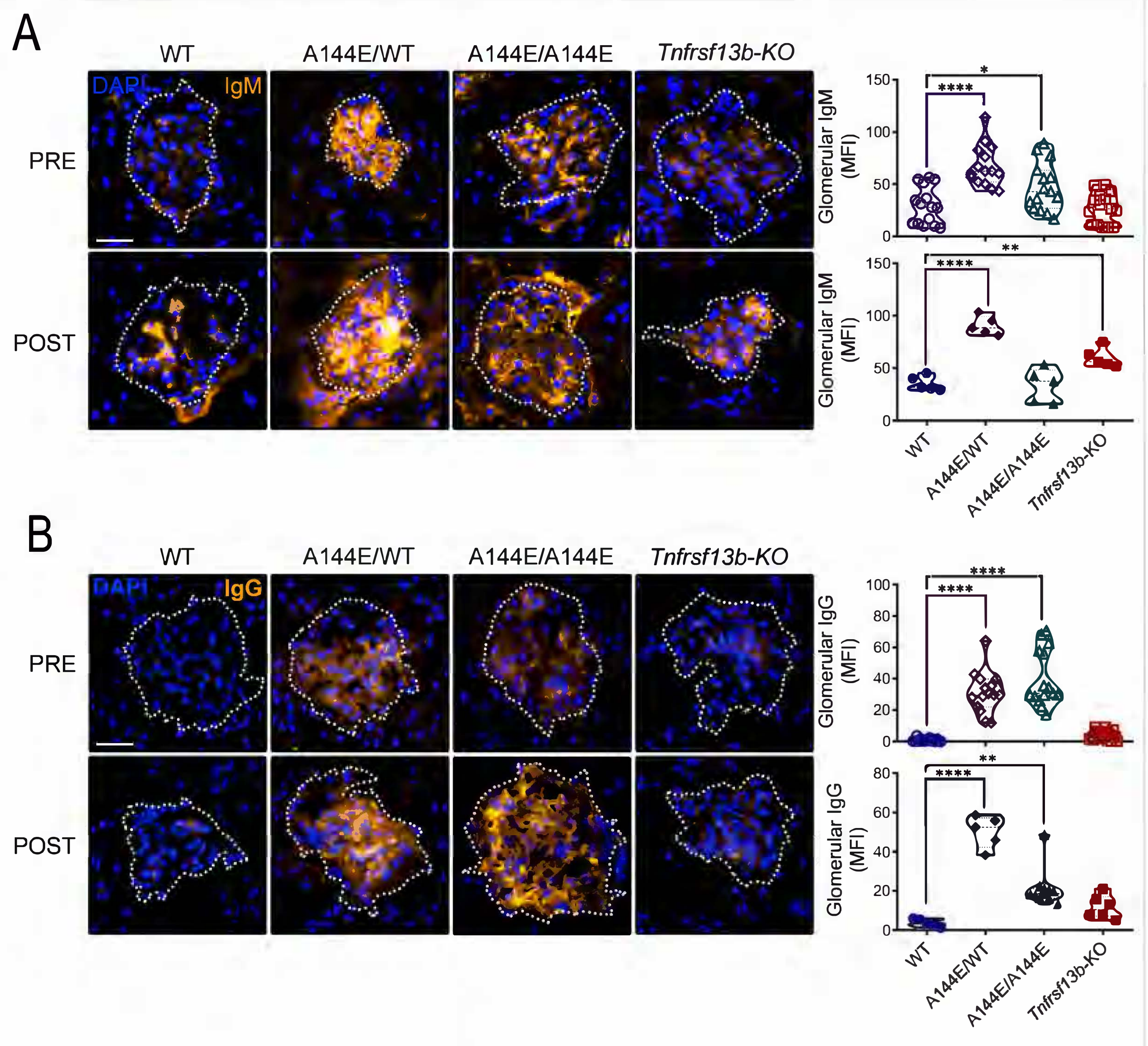

Figure S6. ***Tnfrsf13b*** **protects native kidneys from immune and inflammatory injury. (A and B)** Native kidneys were harvested from naïve mice (pre) or mice immunized via intraperitoneal injection with 5 x 10^7 BALB/c splenocytes and thymocytes (post). Glomerular IgM **(A)** and IgG **(B)** deposits were examined with anti-mouse IgM or IgG immunostaining of frozen native kidney sections pre- or 8 days post-allogeneic stimulation. Bars = 25 µm. Graphs show Mean ± SEM calculated from analysis of 5-7 fields with 3 or more glomeruli per mouse per group. Open shapes represent data from naive mice (pre) and filled shapes represent data of mice 8 days of post-immunization (post). One-Way ANOVA with Dunnett's multiple comparison test or Kruskal-Wallis with Dunn's multiple comparison test: * $p \leq 0.05$; ** $p \leq 0.01$; **** $p \leq 0.0001$ in relation to WT control.

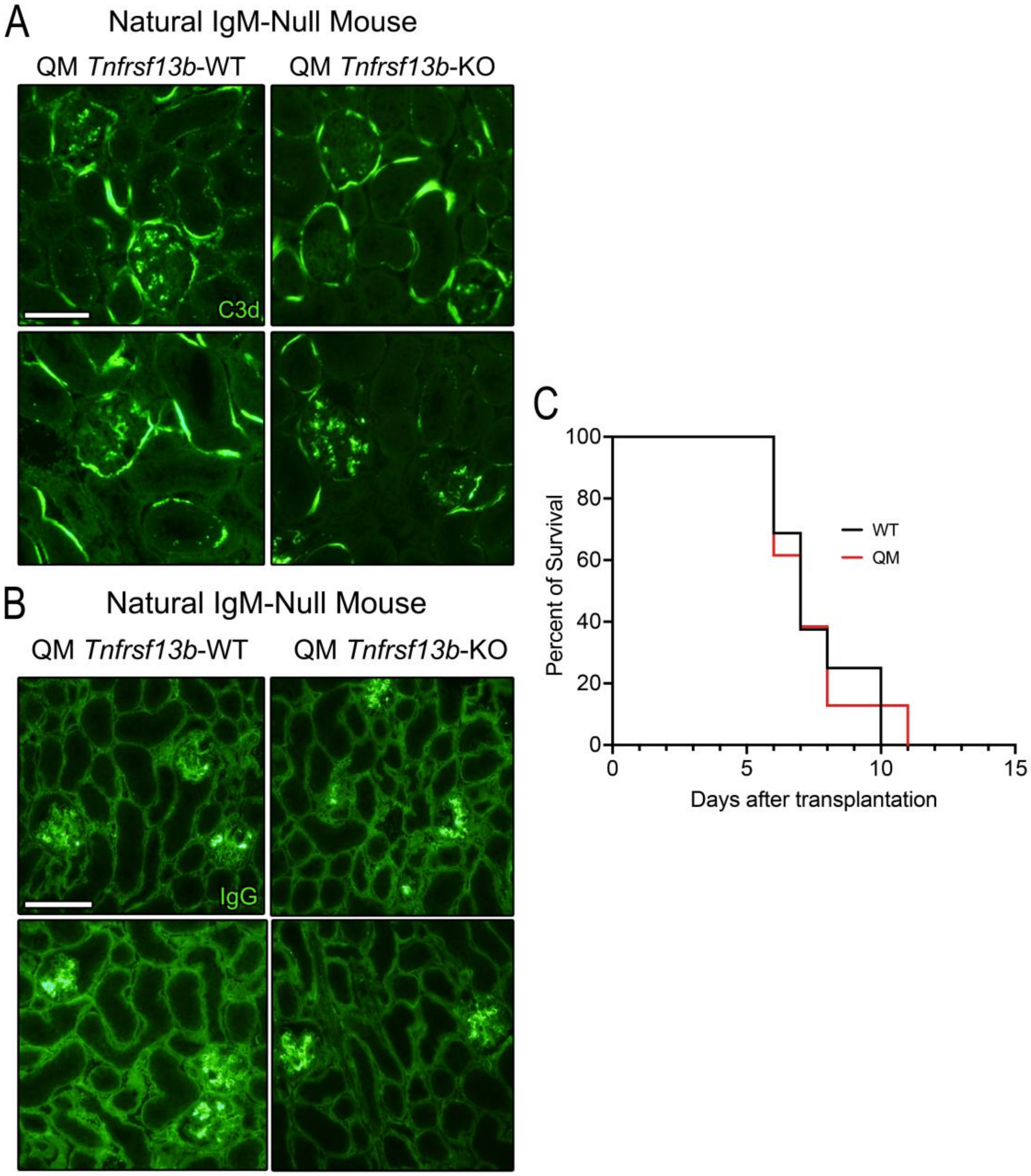

Figure S7. C3d and IgG deposition in native kidneys of natural antibody-deficient mice. Native kidneys were harvested from naive Quasi-Monoclonal (QM) mice *Tnfrsf13b* proficient or deficient. Quasi-Monoclonal mice produce only 4-hydroxy-3-nitrophenyl-acetyl (NP)-specific IgM and lack natural IgM. Glomerular C3d deposits **(A)** or IgG **(B)** were identified with anti-mouse C3d or goat anti-mouse IgG on frozen sections obtained from native kidneys. Figures show typical sections obtained from two distinct *Tnfrsf13b* proficient (left) or deficient (right) Quasi-Monoclonal mice, representative from stainings in 7 different mice of each genetic background. Bar: 25 µm. **(C)** Hearts (transplanted heterotopically into the abdomen) from BALB/c mice H-$2^{d/d}$ haplotype) were transplanted into C57BL/6 (H-$2^{b/b}$ haplotype) WT and QM mice and allograft survival was evaluated daily until rejection. Graph depicts the survival curves showing overlapping rejection kinetics.

Table S1. Predicted impact of *TNFRSF13B* mutations on protein function. Impact of *TNFRSF13B* missense mutations on protein structure and function, according to SIFT, PolyPhen-2, CADD, REVEL, MetaLR and MutationAssessor prediction tools and the ClinVar database.

Exon	Missense Mutation	Variant ID		SIFT		PolyPhen-2		CADD		REVEL		MetaLR		MutationAssessor		ClinVar
		GnomAD Browser	Ensembl													
Exon 3	C104R	17-16852187-A-G	rs34557412	0.000	deleterious	0.999	probably damaging	25	likely benign	0.919	likely disease causing	0.861	damaging	0.828	medium	conflicting interpretations of pathogenicity, risk factor: likely benign (2); likely pathogenic (2); pathogenic (2); uncertain significance (4)
Exon 4	A173T	N.A.	rs369937497	0.160	tolerated	0.030	benign	1	likely benign	0.419	likely benign	0.648	damaging	0.609	medium	N.A.
	A181E	17-16843729-G-T	rs72553883	0.000	deleterious	0.395	benign	10	likely benign	0.607	likely disease causing	0.600	damaging	0.528	medium	conflicting interpretations of pathogenicity: likely benign (1); likely pathogenic (2); pathogenic (2); uncertain significance (1)
	K188del	17-16843705-CTCT-C	rs376630110	-	-	-	-	-	-	-	-	-	-	-	-	benign
	K188M	17-16843708-T-A	rs74811083	0.000	deleterious	0.360	benign	15	likely benign	0.399	likely benign	0.323	tolerated	0.169	neutral	benign/likely benign
	R189M	17-16843705-C-A	rs199777698	0.000	deleterious	0.520	possibly damaging	16	likely benign	0.603	likely disease causing	0.783	damaging	0.528	medium	uncertain significance
	G190R	17-16843703-C-G<br>17-16843703-C-T	rs150101848	0.010	deleterious	0.198	benign	15	likely benign	0.397	likely benign	0.845	damaging	0.609	medium	uncertain significance
	S209F	N.A.	rs1314475404	0.030	deleterious	0.019	benign	16	likely benign	0.326	likely benign	0.692	damaging	0.581	medium	N.A.
Exon 5	P251L	17-16842991-G-A	rs34562254	0.100	tolerated	0.284	benign	12	likely benign	0.261	likely benign	0.000	tolerated	0.261	low	benign

Abbreviations: SIFT, Sorting Intolerant From Tolerant; PolyPhen-2, Polymorphism Phenotyping v2; CADD, Combined Annotation-Dependent Depletion; REVEL, Rare Exome Variant Ensemble Learner: N.A.. Not available.

Table S2. Top 500 germinal center B cell genes differentially expressed between wild type and *Tnfrsf13b*-mutant mice 10 days post-allogenic stimulation.
